# Influence of Smartphone Usage on development of Sexuality among Late Adolescent Boys in Health Sciences Colleges of South India

**DOI:** 10.12688/f1000research.150657.2

**Published:** 2025-01-07

**Authors:** Sharath M Manya, Jayashree K, Prasanna Mithra, Preeti M Galagali, Sara J Ommen

**Affiliations:** 1 Department of Pediatrics, Kasturba Medical College, Mangalore, Manipal Academy of Higher Education, Karnataka, Manipal, 576104, India; 2 Department of Community Medicine, Kasturba Medical College, Mangalore, Manipal Academy of Higher Education,, Karnataka, Manipal, 576104, India; 3 Director and Consultant Adolescent Health, Bangalore Adolescent Care and Counselling Centre, Bangalore, Karnataka, India

**Keywords:** Adolescent boys, sexual orientation, gender identity, smartphone addiction, pornography, problematic smartphone usage

## Abstract

**Background:**

Better affordability of data plans and an increase in “budget” smartphones have resulted in an exponential rise in internet and smartphone users. The ease of access to sexually explicit material (SEM) coupled with adolescents’ impulsivity makes them prone to excessive SEM exposure and may affect the development of sexuality via the perceived realism of such content. This study was done to study the influence between problematic smartphone usage (PSU) and sexuality development among late adolescent boys.

**Methods:**

One hundred and thirty-four adolescents aged 18-19 years, studying in a medical university, participated in this cross-sectional study. Smartphone Addiction Scale – short version (SAS-SV) to evaluate PSU and a content validated semi-structured proforma to evaluate gender identity, sexual orientation, sources of information on pubertal changes, and exposure to pornographic content was used. We expressed results as proportion and summary measures (Mean±SD), Chi-square test to find influence between PSU and adolescent sexuality development.

**Results:**

We found a 45.5% prevalence of PSU. Among study participants, 88% were attracted to the opposite sex, 6% were attracted to the same-sex, and 6% were attracted to both sexes. Three per cent of participants liked wearing clothes of the opposite sex, 7.5% wanted to be members of the opposite sex, whereas 10.4% were not comfortable with their genitalia. They obtained information regarding pubertal changes from friends (85) and media (78). Many had exposure to pornographic content (90%), with the youngest being nine years old.

**Conclusion:**

Nearly half of the late adolescents have PSU. Pornographic contents are accessed through Smartphones.

## Introduction

Adolescence is a stage of transition from childhood to adulthood that includes rapid physical, cognitive, and psychosocial growth. ^1^^,^^2^ It is a critical period, as many behavioral patterns emerge during this period, which may persist throughout life. ^3^ Psychological well-being and overall health of adolescents are major concerns worldwide. ^4^ Adolescents engage in life-threatening behaviors, such as alcohol and substance use, unsafe sex, poor eating, and heightened risk-taking, which affects their psychological well-being. ^4^^,^^5^ According to the 2011 census, in India, 1/4 ^th^ of the population are adolescents (253 million), and 54% belong to the 10-14 age group, and nearly 46% are in the 15-19 years of age. ^6^

Adolescents are highly attracted to modern technologies because they are psycho-socially immature and more prone to smartphone addiction due to the influence of the environment and peers. ^7^ Reports highlight that adolescents are highly addicted to smartphones, equivalent to substances and various types of behavioral addiction. ^8^ A meta-analysis by Davey et al. showed prevalence of smartphone abuse among Indian adolescents to be approximately 39% - 44%. ^9^ Billieux et al. documented that problematic smartphone use (PSU) is defined as “an inability to regulate one’s use of the smartphone, which eventually involves negative consequences in daily life”. ^10^ It is a behavioral or psychological dependence on mobile devices that is closely linked to excessive digital media use, such as Internet addiction disorder. ^11^ PSU has been considered as a measure of smartphone addiction and is associated with various psychopathologies. ^12^

Adolescents with PSU are highly exposed to viewing sexualized content through videos and reality programs. In addition to these sources, social media platforms and various mobile applications have increased sexual content. ^13^ The adolescents are more frequently engaged in online sex chat, and are thereby exposed to cyberbullying and online harassment. ^14^ PSU may lead to impulsiveness, which plays a pivotal role in an increased number of sexual partners. ^15^

The term sexual orientation refers to the sex to which individuals are sexually or romantically attracted. Gender identity refers to an individual’s basic sense of being male, female, transgender, or something else. ^16^ Gender orientation is biologically determined, and sexual expressions have many external influences, such as the media. For the present study, the identification of gender roles, orientation and identity was considered as the development of sexuality based on previous works in literature. ^17^ However, there is a paucity of information on how PSU affects sexuality in adolescence. The present study aimed to understand the existing patterns of smartphone usage among late adolescents and their association with the development of adolescent male sexuality.

## Methods

### Study design & participants

We conducted a cross-sectional study among male students pursuing medical, dental, and allied health sciences courses aged between 18 and 19 years at a medical university in Mangalore, an urban area in the South Indian State of Karnataka. Late adolescent boys who were unwilling to participate were termed non-responders and were excluded from the study. The study duration was 11 months (November 2019–September 2020). We followed a convenience sampling method to recruit study participants. The STROBE guidelines were adhered to, and a complete checklist was provided in the reporting guidelines.

### Sample size

**Figure 1.**
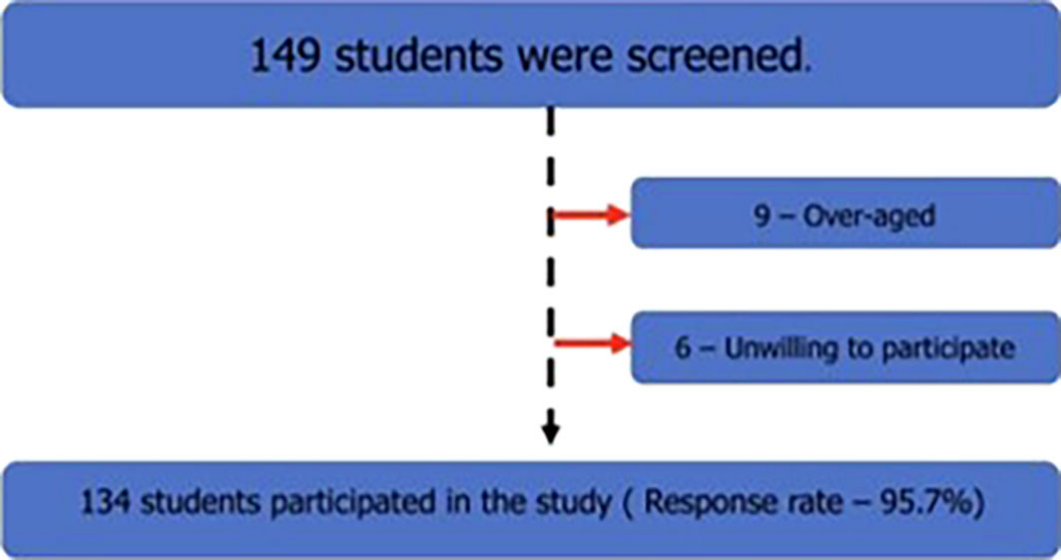
The study flow diagram.

All male students aged between 18 and 19 years who pursued the above-mentioned courses in the study institute were screened, and those over age and unwilling to participate were excluded from the study. The study flow diagram is shown in the following Figure 1:

### Study tool

Following the consenting process, a content-validated semi-structured questionnaire (Google Form) was electronically mailed to all participants who were willing to participate in the study, and responses were collected. Students who had not completed the Google form were reminded using a WhatsApp message (24 hours after sending the questionnaire) and telephonic conversation (48 hours after sending the questionnaire). The questionnaire included demographic details, The Smartphone Addiction Scale – short version (SAS-SV), ^18^ and questions on gender identity, sexual orientation, sources of information regarding pubertal changes, and body image issues. Smartphone usage and addiction using SAS-SV developed by Kwon et al., ^18^ consists of 10 items with 6 point Likert type scale. The scale items were scored from 1 to 6, with 1 being ‘strongly disagree’ and 6 being ‘strongly agree.’ The total possible score was between 10 and 60, with higher scores indicating higher chances of smartphone addiction. Based on the findings of a previous study, the cut-off for smartphone addiction was determined to be 31 in the present study. ^18^

### Data analysis

Data analysis was performed using IBM SPSS Statistics for Windows, Version 25.0. Armonk, NY: IBM Corp. Results are expressed as proportions and summary measures (mean ± standard deviation) using appropriate tables and figures. The influence between smartphone usage and sexual development was assessed using the chi-square test and independent sample t-test. Statistical significance was set at P <0.05.

## Results

A total of 149 students were screened, and nine were excluded from the study because they were older. Among the remaining 140 students, 6 were unwilling to participate in this study; hence, they were excluded. The response rate was 95.7%. Of the 134 students, 98 were medical students, 20 were dental students, and 16 were allied health sciences students. Among the students, 90.3% were permanent residents of India, followed by 6.7% nonresidential Indians, and 3% foreign nationals. Most of the students resided in the hostel (85.1%), while 13.4% of the students lived at home, and 1.5% stayed in an apartment with friends.

In the present study, based on a predetermined cut off score of 31 on SAS-SV, the prevalence of problematic smartphone use (PSU) was 45.5% among the students.

**Figure 2.**
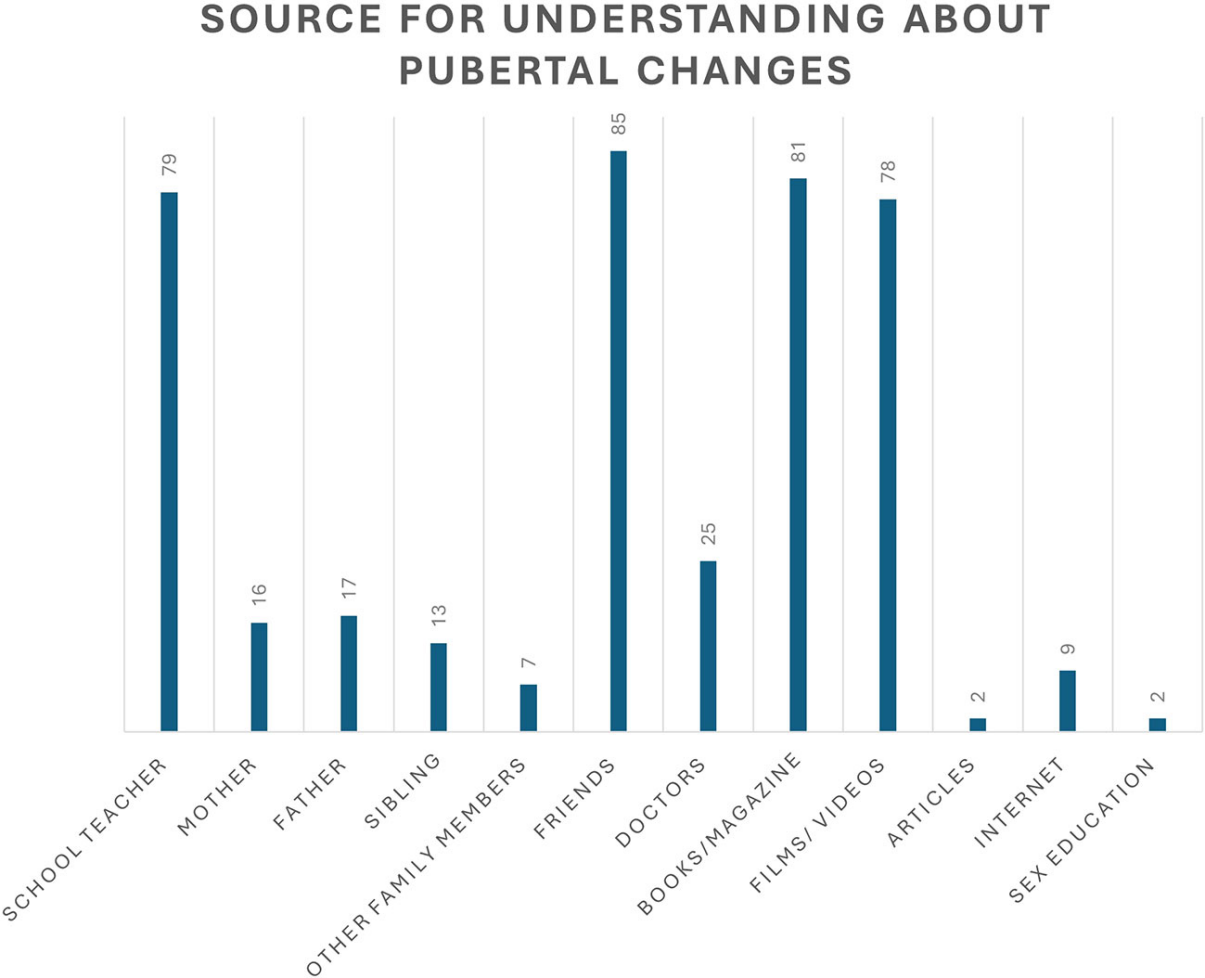
Source of understanding about pubertal changes.

As shown in Figure 2, 85 students obtained information about pubertal changes from friends, followed by books/magazines (81), schoolteachers (79), and movies (78). In contrast, fewer adolescents turned to mothers (n=16), fathers (n=17), or doctors (n=18) for information related to pubertal changes.

As shown in Table 1, regarding sexual orientation and gender identity among the students, 3% of the students desired to wear the dress of the opposite sex, while 94.8% did not, and 2.2% were not unsure. Among these students, 87.3% were happy with their current sexual identity, while 7.5% of them wanted to be members of the opposite sex. 86.6% Of students were comfortable with their genitalia, whereas 10.4% were unsatisfied. Romantic feelings towards the opposite sex were perceived to be normal by 91.8% of the students, while 5.2% felt abnormal, and 3% were not unsure. Out Of these students, 88% were attracted to the opposite sex, while 6% were attracted to the same sex, and 6% were attracted to both sexes.

**Table 1.** Smart phone usage and body characteristics of the study participants (n=134).

Are you worried about your:	Responses	Smart phone usage *		P value
		<31 No. (%)	>31 No. (%)	
Physique	No	27 (36.9)	23 (37.7)	0.932
	Yes	46 (63.1)	38 (62.3)	
Weight	No	35 (47.9)	24 (39.3)	0.318
	Yes	38 (52.1)	37 (60.7)	
Height	No	52 (71.2)	47 (77.0)	0.445
	Yes	21 (28.8)	14 (23.0)	
Skin colour	No	59 (80.8)	49 (80.3)	0.943
	Yes	14 (19.2)	12 (19.7)	
Scalp hair	No	46 (63.0)	34 (55.7)	0.392
	Yes	27 (37.0)	27 (44.3)	
Body hair	No	59 (80.8)	55 (90.2)	0.131
	Yes	14 (19.2)	06 (09.8)	
Optic aid	No	60 (82.2)	49 (80.3)	0.783
	Yes	13 (17.8)	12 (19.7)	
Genitals	No	66 (90.4)	51 (83.6)	0.239
	Yes	07 (09.4)	10 (16.4)	

* ≥31 on SAS-SV scale was considered problematic for smartphone use.

Table 2 shows no significant association between PSU and gender identity or PSU and sexual orientation. Regarding the knowledge of adolescent sexuality, 72% attended classes on the topics related to sex in schools, 19% had not attended, and 9% were not sure.

**Table 2.** Gender identity and sexual orientation (n=134).

Responses	Yes n (%)	No n (%)	Don’t know n (%)
Do you like wearing clothes of the opposite sex?	04 (03.0)	127 (94.8)	03 (02.2)
Would you like to be a member of the opposite sex?	10 (07.5)	117 (87.3)	07 (05.2)
Do you feel uncomfortable with your genitalia?	14 (10.4)	116 (86.6)	04 (03.0)
Do you feel romantic feeling towards the opposite sex is normal?	123 (91.8)	07 (05.2)	04 (03.0)
Do you have attraction/feeling towards another person of the opposite sex?	123 (91.8)	04 (03.0)	07 (05.2)
Do you have attraction/feeling towards another person of the same-sex?	08 (06.0)	119 (88.8)	07 (05.2)
Do you have attractions/feelings toward people of both sexes?	08 (06.0)	114 (85.1)	12 (09.0)

Regarding normal male sexuality, 92.5% of the students were aware of the mental changes occurring during puberty, 90.3% were aware that adolescence was the final opportunity for growth (final height), and 83.6% of the study population indulged in masturbation. Among the adolescents indulged in masturbation, 28% felt guilty after masturbation, 30% felt masturbation caused severe damage to health, and 99.1% of the students preferred masturbation in private and thought it was not normal to masturbate in public. There was no statistically significant association between PSU on gender identity and sexual orientation ( Table 3)

**Table 3.** Effect of PSU on Gender Identity and sexual orientation (n=134).

Responses		Problematic smartphone usage		P value
		No n (%)	Yes n (%)	
Liking wearing clothes of the opposite sex	No	71 (54.6)	59 (45.4)	1
	Yes	02 (50.0)	02 (50.0)	
Liking to be a member of the opposite sex	No	69 (55.6)	55 (44.4)	0.511
	Yes	04 (40.0)	06 (60.0)	
Feeling uncomfortable with own genitalia	No	65 (54.2)	55 (45.8)	1
	Yes	08 (57.1)	06 (42.9)	
Feeling that romantic feeling towards the opposite sex is normal	No	08 (72.7)	03 (27.3)	0.344
	Yes	65 (52.8)	58 (47.1)	
Having attraction/feeling towards another person of the opposite sex	No	06 (54.5)	05 (45.5)	1
	Yes	67 (54.5)	56 (45.5)	
Having attraction/feeling towards another person of the same sex	No	67 (53.2)	59 (46.8)	0.290
	Yes	06 (75.0)	02 (25.0)	
Having attractions/feelings toward people of both sexes	No	66 (53.7)	60 (46.3)	0.071
	Yes	07 (87.5)	01 (12.5)	

Statistical significance was set at P <0.05.

.

In our study, 90% students had watched pornographic content and one of the students had watched when he was 9 years old, 4.2% watched porn content before they turned 10, and 58% watched the porn content between 15-18 years. Most of the students (95%) wanted to watch the porn content alone, and 90.8% watched the porn content through smartphones. Regarding the frequency of watching porn content, 45% viewed less than once a week, and 37% watched 2-3 times per a week.

After watching the pornographic content, the psychological behaviour shows that 7.5% of the students felt altered perception of communicating with the opposite sex, 11.7% responded that watching porn content had altered the study pattern, and 12.5% answered that it affected their academic performance. However, 71% of the students revealed that they had been sexting at least once. Our study did not find a statistically significant association between PSU and exposure to pornographic content ( Table 4).

**Table 4.** Association between smartphone usage and pornography.

Responses		Problematic smartphone usage		P value
		No (n=66) n (%)	Yes (n=54) n (%)	
At what age did you first watch a pornographic movie (PORN)? Mean (SD)		14.43 (1.99)	15.16 (2.16)	0.056
How frequently do you watch PORN?	≤once a week	29 (43.9)	25 (46.3)	0.841
	≥once a day	07 (10.6)	04 (07.4)	
	2-3 times a week	23 (34.8)	21 (38.8)	
	once a day	7 (10.6)	04 (07.4)	
Does it affect academic performance?	No	60 (90.9)	46 (85.2)	0.331
	Yes	6 (9.1)	8 (14.8)	

## Discussion

Sexuality, which involves showing interest in a desired activity, orientation, and preference, is a normal phenomenon during adolescence. Adolescent sexuality includes an array of factors, such as intimate partnerships, gender identity, sexual orientation, religion, and culture. ^19^ Adolescent sexuality development correlates better with the biopsychosocial model. ^20^ The array of biological, psychological, and social factors plays a predominant role in the progression of adolescent sexuality. Social factors such as parents’ attitudes related to sexuality, parenting style, relationship with peers, and cultural influence are the major attributes of adolescent sexual attitudes. ^21^ Recently, the media have also greatly influenced adolescent sexuality. Adolescents have easy access to information on sexuality, sex crimes, and violence through the media, influencing their perceptions and attitudes toward sexuality. ^22^ Further, television and the Internet also provide fast access to sexual content, altering adolescents’ perception towards sexuality. ^23^ Smartphone provides various benefits such as fast internet access, video calling, easy access to social media, and gaming. ^24^ Increased smartphone use leads to addictive behavior. One commonly referred term is Problematic Smartphone Use (PSU), similar to behavioral addictions such as substance-related, gambling, and Internet addiction disorder. ^25^ Adolescents are more vulnerable to problematic smartphone use because of the imbalance between the reward and behavioral control systems. ^18^ A recent meta-analysis showed that Indian adolescents are highly addicted to smartphones and exhibit problematic sexual behaviors. ^9^ So, we conducted the present study to evaluate the impact of problematic smartphone use on male adolescent sexuality development. The late adolescent stage is the period of sexual orientation stability and also development to maturity towards responding to various aspects of the gender-related issue, smartphone usage; we chose the adolescents in 18-19 years age group.

The prevalence of smartphone addiction based on the SAS-SV cut-off score of 31 was found to be 45.5%, which reflects a higher PSU as compared to Chinese students (38.5%), ^26^ European countries Belgium (21.5%) ^25^ and American university students (20.1%). ^15^ This discrepancy might be due to differences in sampling methods and the rapid development of telecommunication with cheaper and faster internet along with an increase in the budget of smartphones in India. ^27^

In our study, transvestism, is observed in 3% of the students. Among the participants, 10.4% reported dissatisfaction with their genitalia. Men’s attitudes toward genitals have been shown to affect their psychological status. Thus, in men with dissatisfied genitals, it creates a state of insecurity, and in men with contented genitals, it gives confidence. ^28^ The majority of the participants, 91.8%) thought romantic feelings towards their female counterparts were normal. Romantic relationships during adolescence are essential for development and well-being. Further, it has a positive association with the development of personal identity, self-esteem, and social competence and is useful for career planning. It has been positively associated with the formation of personal identity, adaptability to change, self-esteem, social competence, scholastic achievement, and career planning. ^29^ Late adolescence (16–18 years) occurs when consolidation of romantic bonds occurs, elevating the risk of a poor-quality romantic relationship. ^30^ In our study, normal romantic relationships towards the opposite sex might be due to a greater preference for cyber-relationship applications such as web chats and social networking chat rooms. These social networking chat rooms offer online romantic relations, which might be heightened by excessive or problematic internet use. ^31^ We report 6% of same-sex attraction among the students; however, in a study conducted by Nama et al. among Canadian medical students in Ottawa, 18% of the medical students were involved in same-sex attraction or gay behavior. ^32^ Further, our study reported no significant associations between sexual orientation, gender identity, and PSU. In this study, a higher number of students (85%) obtained information regarding the pubertal changes from their friends. Previous studies have also revealed that males share information regarding puberty and first wet dreams with their friends. ^33^

In this study, 72% of adolescents attended sex education in schools. In a study done by Kumar et al. shows that 97.1% of students like to have sex education during their school life period. ^34^ Further, genital growth in boys has been reported to be associated with altered cognitive function. ^35^ In this study, 92.5% of students were aware of mental changes during puberty, 90.3% aware that adolescence is a final opportunity for growth, and 83.6% of students had self-explored genitalia.

In this study, 28% of students felt guilty after masturbation, 30% felt masturbation could induce serious damage to health, and 99.1% preferred private masturbating practice and not in public. Similar to our report, a study conducted among Chinese students showed an association between floating anxiety, somatic and hysterical symptoms, and masturbation. ^2^

In our study, 90% of the students had viewed porn content, which is comparable to other studies conducted among adolescent boys in Sweden (98%) ^36^ and Australia (87%). ^37^ In the current study, 45% viewed porn content less than once a week, and 37% watched 2-3 times per a week, which is higher as compared to a study done among Polish students, where 34% viewed pornographic content once a week and 24% few times a week. ^38^

In this study, watching pornographic content did not influence students’ psychological behavior and study patterns. Similar to our report, a study conducted on Croatian adolescents showed that viewing pornography displayed no significant negative alteration in adolescent subjective well-being, depression or anxiety, or self-esteem. ^39^ In a study conducted by Šević et al. among Croatian adolescents, watching porn content or sexually explicit material online did not affect academic grades, study patterns, or well-being. ^40^ In contrast, Beyens et al. reported that watching online pornographic content has a negative association with students’ academic performance when compared between baseline and six months. ^41^ Further, we did not observe a significant association between problematic smartphone use and pornographic content exposure.

The strength of this study is that it is the first of its kind to correlate adolescent sexuality with smartphone usage using a standardized questionnaire (Smartphone Addiction Scale – short version, SAS-SV). This study has some limitations. Considering the smaller sample size and enrolment of only health sciences students, the results cannot be generalized.

This study implies the formulation of structured awareness programs to be carried out by health professionals and to educate parents about handling adolescent issues. In addition, it encourages adolescent health programs to create knowledge about risky behaviors and community support groups. This study highlights the need to include comprehensive sex education as part of the school curriculum and to educate young Internet users on the safe and responsible use of the Internet.

## Ethics and consent

The study was approved by the Institutional Ethics Committee on 15/01/2020 (approval number: IEC KMC MLR 01/2020/36) and the Head of the Institute. Eligible participants were contacted individually using the MS Teams platform. The purpose of the study, the role of their participation, the study procedure, and confidentiality were explained, and informed consent was obtained online using google forms from each participant.

## Data Availability

Open Science Framework: Influence of Smartphone Usage on the development of sexuality among Late Adolescent Boys in Health Sciences Colleges of South India. https://doi.org/10.17605/OSF.IO/KVM4A ^42^ This dataset contains the following underlying data:
• Excel Data file Open Science Framework: Influence of Smartphone Usage on development of Sexuality among Late Adolescent Boys in Health Sciences Colleges of South India. https://doi.org/10.17605/OSF.IO/KVM4A ^42^ This dataset contains the following underlying extended data:
• Participant information sheet. • Informed consent form • Study Questionnaire Data are available under the terms of the Creative Commons Attribution 4.0 International license (CC-BY 4.0) Open Science Framework: Influence of Smartphone Usage on development of Sexuality among Late Adolescent Boys in Health Sciences Colleges of South India. https://doi.org/10.17605/OSF.IO/KVM4A ^42^ It contains the STROBE checklist for the Influence of Smartphone Usage on the development of sexuality among Late Adolescent Boys in Health Sciences Colleges of South India. The pre-print version of this study is available online from: https://doi.org/10.21203/rs.3.rs-1800750/v1 ^43^

## References

[1] Sexual and Reproductive Health and Research (SRH):[cited 2024 Apr 6]. Reference Source

[2] Jiao T Chen J Niu Y : Masturbation is associated with psychopathological and reproduction health conditions: an online survey among campus male students. *Sex. Relatsh. Ther.* 2019;37:272–286. 10.1080/14681994.2019.1677883

[3] Mawson E Best D Beckwith M : Social identity, social networks and recovery capital in emerging adulthood: A pilot study. *Subst. Abuse Treat. Prev. Policy.* 2015;10(1):45. 10.1186/s13011-015-0041-2 26560076 PMC4642657

[4] Salam RA Das JK Lassi ZS : Adolescent health and well-being: Background and methodology for review of potential interventions. *J. Adolesc. Health.* 2016;59(4):S4–S10. 10.1016/j.jadohealth.2016.07.023 27664594 PMC5026682

[5] Balogh KN Mayes LC Potenza MN : Risk-taking and decision-making in youth: Relationships to addiction vulnerability. *J. Behav. Addict.* 2013;2(1):1–9. 10.1556/JBA.2.2013.1.1 24294500 PMC3840427

[6] Census of India Website: Office of the Registrar General & Census Commissioner, India:[cited 2019 Nov 27]. Reference Source

[7] Nahas M Hlais S Saberian C : Problematic smartphone use among Lebanese adults aged 18–65 years using MPPUS-10. *Comput. Hum. Behav.* 2018 Oct 1;87:348–353. 10.1016/j.chb.2018.06.009

[8] Jo H Na E Kim D-J : The relationship between smartphone addiction predisposition and impulsivity among Korean smartphone users. *Addict. Res. Theory.* 2018;26(1):77–84. 10.1080/16066359.2017.1312356

[9] Davey S Davey A : Assessment of smartphone addiction in Indian adolescents: a mixed method study by systematic-review and meta-analysis approach. *Int. J. Prev. Med.* 2014;5(12):1500–1511. 25709785 PMC4336980

[10] Billieux J Maurage P Lopez-Fernandez O : Can disordered mobile phone use be considered a behavioral addiction? An update on current evidence and a comprehensive model for future research. *Curr. Addict. Rep.* 2015;2(2):156–162. 10.1007/s40429-015-0054-y

[11] Kuss DJ Harkin L Kanjo E : Problematic smartphone use: Investigating contemporary experiences using a convergent design. *Int. J. Environ. Res. Public Health.* 2018;15(1):142. 10.3390/ijerph15010142 29337883 PMC5800241

[12] Costa R Martins C Fuzeiro V : Sexual function and problematic use of smartphones and online social networking sites. *J. Sex. Med.* 2022;19(5):S153. 10.1016/j.jsxm.2022.03.352 35718741

[13] Peter J Valkenburg PM : Adolescents and pornography: A review of 20 years of research. *J. Sex Res.* 2016;53(4–5):509–531. 10.1080/00224499.2016.1143441 27105446

[14] Matković T Cohen N Štulhofer A : The use of sexually explicit material and its relationship to adolescent sexual activity. *J. Adolesc. Health.* 2018;62(5):563–569. 10.1016/j.jadohealth.2017.11.305 29503032

[15] Grant JE Lust K Chamberlain SR : Problematic smartphone use associated with greater alcohol consumption, mental health issues, poorer academic performance, and impulsivity. *J. Behav. Addict.* 2019;8(2):335–342. 10.1556/2006.8.2019.32 31257917 PMC6609450

[16] Sprott RA Anhalt K Goodenow C : *Resolution on Gender and Sexual Orientation Diversity in Children and Adolescents in Schools.* American Psychological Association & National Association of School Psychologists;2015 [cited 2020 Oct 27]. Reference Source

[17] Kar SK Choudhury A Singh AP : Understanding normal development of adolescent sexuality: A bumpy ride. *J. Hum. Reprod. Sci.* 2015;8(2):70–74. 10.4103/0974-1208.158594 26157296 PMC4477452

[18] Kwon M Kim D-JJ Cho H : The smartphone addiction scale: Development and validation of a short version for adolescents. *PLoS One.* 2013;8(12):1–7. 10.1371/journal.pone.0083558 PMC3877074 24391787

[19] Sales JM Smearman EL Brody GH : Factors associated with sexual arousal, sexual sensation seeking and sexual satisfaction among female African American adolescents. *Sex. Health.* 2013;10(6):512–521. 10.1071/SH13005 24262218 PMC3839054

[20] Merrick J Tenenbaum A Omar HA : Human sexuality and adolescence. *Front. Public Health.* 2013;1:41. 24350210 10.3389/fpubh.2013.00041 PMC3859969

[21] Harris AL : Media and technology in adolescent sexual education and safety. *J. Obstet. Gynecol. Neonatal. Nurs.* 2011;40(2):235–242. 10.1111/j.1552-6909.2011.01218.x 21284726

[22] Scull TM Malik CV : Role of entertainment media in sexual socialization. *Int. Encycl. Media Lit.* 2019;1–11. 10.1002/9781118978238.ieml0214

[23] Horvath J Mundinger C Schmitgen MM : Structural and functional correlates of smartphone addiction. *Addict. Behav.* 2020;105:106334. 10.1016/j.addbeh.2020.106334 32062336

[24] Lin Y-H Chang L-R Lee Y-H : Development and validation of the Smartphone Addiction Inventory (SPAI). *PLoS One.* 2014;9(6):e98312. 10.1371/journal.pone.0098312 24896252 PMC4045675

[25] Steinberg L Albert D Cauffman E : Age differences in sensation seeking and impulsivity as indexed by behavior and self-report: evidence for a dual systems model. *Dev. Psychol.* 2008;44(6):1764–1778. 10.1037/a0012955 18999337

[26] Luk TT Wang MP Shen C : Short version of the Smartphone Addiction Scale in Chinese adults: Psychometric properties, sociodemographic, and health behavioral correlates. *J. Behav. Addict.* 2018;7(4):1157–1165. 10.1556/2006.7.2018.105 30418073 PMC6376375

[27] Cable.co.uk: Worldwide mobile data pricing: The cost of 1GB of mobile data in 230 countries. 2020 [cited 2020 Aug 3]. Reference Source

[28] Lever J Frederick DA Peplau LA : Does size matter? Men’s and women’s views on penis size across the lifespan. *Psychol. Men Masculinity.* 2006;7(3):129–143. 10.1037/1524-9220.7.3.129

[29] Collins WA Welsh DP Furman W : Adolescent romantic relationships. *Annu. Rev. Psychol.* 2009;60:631–652. 10.1146/annurev.psych.60.110707.163459 19035830

[30] Luciano EC Orth U : Transitions in romantic relationships and development of self-esteem. *J. Pers. Soc. Psychol.* 2017;112(2):307–328. 10.1037/pspp0000109 27379474

[31] Anderson EL Steen E Stavropoulos V : Internet use and Problematic Internet Use: A systematic review of longitudinal research trends in adolescence and emergent adulthood. *Int. J. Adolesc. Youth.* 2017;22(4):430–454. 10.1080/02673843.2016.1227716

[32] Nama N MacPherson P Sampson M : Medical students’ perception of lesbian, gay, bisexual, and transgender (LGBT) discrimination in their learning environment and their self-reported comfort level for caring for LGBT patients: a survey study. *Med. Educ. Online.* 2017;22(1):1368850. 10.1080/10872981.2017.1368850 28853327 PMC5653936

[33] Aktar B Sarker M Jenkins A : Exploring adolescent reproductive health knowledge, perceptions, and behavior among students of non-government secondary schools supported by the BRAC mentoring program in rural Bangladesh. *J. Asian Midwives.* 2014;1(1):17–33.

[34] Kumar R Goyal A Singh P : Knowledge attitude and perception of sex education among school going adolescents in Ambala district, Haryana, India: a cross-sectional study. *J. Clin. Diagnostic Res. JCDR.* 2017;11(3):LC01. 10.7860/JCDR/2017/19290.9338 PMC5427339 28511413

[35] Blakemore S Choudhury S : Development of the adolescent brain: implications for executive function and social cognition. *J. Child Psychol. Psychiatry.* 2006;47(3-4):296–312. 10.1111/j.1469-7610.2006.01611.x 16492261

[36] Donevan M Mattebo M : The relationship between frequent pornography consumption, behaviours, and sexual preoccupancy among male adolescents in Sweden. *Sex. Reprod. Healthc. Off. J. Swedish Assoc. Midwives.* 2017;12:82–87. 10.1016/j.srhc.2017.03.002 28477937

[37] Lim MSC Agius PA Carrotte ER : Young Australians’ use of pornography and associations with sexual risk behaviours. *Aust. N. Z. J. Public Health.* 2017;41(4):438–443. 10.1111/1753-6405.12678 28664609

[38] Dwulit AD Rzymski P : Prevalence, patterns and self-perceived effects of pornography consumption in Polish university students: a cross-sectional study. *Int. J. Environ. Res. Public Health.* 2019;16(10):1861. 10.3390/ijerph16101861 31137778 PMC6571756

[39] Kohut T Štulhofer A : Is pornography use a risk for adolescent well-being? An examination of temporal relationships in two independent panel samples. *PLoS One.* 2018;13(8):e0202048. 10.1371/journal.pone.0202048 30096173 PMC6088458

[40] Šević S Mehulić J Štulhofer A : Is pornography a risk for adolescent academic achievement? findings from two longitudinal studies of male adolescents. *Eur. J. Dev. Psychol.* 2020;17(2):275–292. 10.1080/17405629.2019.1588104

[41] Beyens I Vandenbosch L Eggermont S : Early adolescent boys’ exposure to Internet pornography: Relationships to pubertal timing, sensation seeking, and academic performance. *J. Early Adolesc.* 2015;35(8):1045–1068. 10.1177/0272431614548069

[42] Manya S Jayashree K Mithra P : Influence of smartphone usage on development of sexuality among late adolescent boys in health sciences colleges of South India.(Dataset).2024. Reference Source 10.12688/f1000research.150657.2 PMC11757915 39866731

[43] Manya S Jayashree K Mithra P : Influence of smartphone usage on development of sexuality among late adolescent boys in health sciences colleges of south India. *Research square.* 2022. Reference Source 10.12688/f1000research.150657.2 PMC11757915 39866731

