# Supplementary material for: Influence of Smartphone Usage on development of Sexuality among Late Adolescent Boys in Health Sciences Colleges of South India
Source: F1000Res. 2025 Jan 7;13:1079. Originally published 2024 Sep 20. [Version 2] doi: 10.12688/f1000research.150657.2 (PMC11757915; doi:10.12688/f1000research.150657.2)
Supplement: Peer review history

# Reviewer report: Reviewer response for version 2

**DOI:** 10.5256/f1000research.176146.r361743

**Published:** 2025-01-23

**Authors:** Anjan Mandara

**Affiliations:** 1 Consultant adolescent psychiatrist, Cheshire and Wirral Partnership NHS Foundation Trust, Chester, UK

The article addresses the impact of smartphone usage on sexuality development in young people attending health and allied courses in an urban city. It is a cross sectional study carried out on biological males attending college and the response rate for the cohort was high. A standardised questionnaire was used to collect data and Smartphone Addiction Scale ( SAS-VS) was used to evaluate smartphone use. SAS-VS has got good reliability and validity for assessment of smartphone addiction in community and research settings. Appropriate statistical analysis tool was used to analyse the collected data. The conclusions drawn from the study were compared against published literature and limitations from the study were identified.

 The participants of the study were biological male students enrolled in health and allied health courses in Mangalore between the ages of 18 and 19. The authors have identified that sample size and sample source are limiting factors for the study. It would have been beneficial to invite all students irrespective of their biological sex or gender be invited to take part in the study. I did not have access to the questionnaire developed by the study team or understanding of the process followed to develop it. It would have been useful to see the content of the questionnaire. Based on the results from the data collection, I noted that there was no specific mention of sexual orientation like pan sexual or asexual taken into consideration in the questionnaire.

 Major points

 None

 Minor points

 The authors may wish to develop further on development of gender identity and sexual orientation. For eg. Most people develop and understanding and know their gender by age 3-5 years where as sexual orientation is more a developmental process.

 Authors may consider commenting on impact of social and communication difficulties ( pervasive developmental disorder) and sexual orientation

 Authors may wish to comment/consider sexual orientations not described in paper like pansexual and asexual.

 Authors may wish to share the questionnaire they used to collect the data.

Is the work clearly and accurately presented and does it cite the current literature?

Yes

If applicable, is the statistical analysis and its interpretation appropriate?

Yes

Are all the source data underlying the results available to ensure full reproducibility?

Yes

Is the study design appropriate and is the work technically sound?

Yes

Are the conclusions drawn adequately supported by the results?

Yes

Are sufficient details of methods and analysis provided to allow replication by others?

Yes

Reviewer Expertise:

NA

I confirm that I have read this submission and believe that I have an appropriate level of expertise to confirm that it is of an acceptable scientific standard.

# Reviewer report: Reviewer response for version 2

**DOI:** 10.5256/f1000research.176146.r356982

**Published:** 2025-01-09

**Authors:** Sudhir Prabhu

**Affiliations:** 1 Department of Community Medicine, Father Muller Medical College, Mangalore, India

The authors have carefully considered and adequately addressed all of the scientific and technical concerns raised in the previous review. Based on the author responses, I believe the manuscript has been significantly improved.

Is the work clearly and accurately presented and does it cite the current literature?

Yes

If applicable, is the statistical analysis and its interpretation appropriate?

Yes

Are all the source data underlying the results available to ensure full reproducibility?

Yes

Is the study design appropriate and is the work technically sound?

Yes

Are the conclusions drawn adequately supported by the results?

Yes

Are sufficient details of methods and analysis provided to allow replication by others?

Partly

Reviewer Expertise:

Immunization, Adolescent health, Primary health care

I confirm that I have read this submission and believe that I have an appropriate level of expertise to confirm that it is of an acceptable scientific standard.

# Reviewer report: Reviewer response for version 1

**DOI:** 10.5256/f1000research.165247.r326604

**Published:** 2024-10-11

**Authors:** Sudhir Prabhu

**Affiliations:** 1 Department of Community Medicine, Father Muller Medical College, Mangalore, India

The research article submitted by the authors clearly answers the research question about the impact of problematic smartphone usage and sexuality among late adolescent males, both of which are important public health issues. However I have a few concerns about the study which I would seek clarification from the authors :

 Major :

 1. How was development of sexuality defined for this descriptive study : was it sexual orientation or sexual identity or something else ?

 2. Sexual awareness may begin at early adolescents (9-12 yrs) , what was the reason authors tried to address or justify development of sexuality or influence of smartphone on sexuality at the age of 18-19 yrs ? Was this based on any observations or references or any other issues specific in your region?

 3. Discussion section mentions about friends/peers being the major source for information, but results also show magazines and videos also which may be due to stigma to discuss with teachers, family or health professionals. Kindly elaborate this as it is relevant for the study objective with smartphone being the most easily handy and private source for all information for an adolescent.

 Minor

 1. Abstract mentions about "exposure to pornographic content with youngest being 9 years old" study subjects being late adolescents does this mean at the age of 9 ?

 2. Are smartphone addiction and problematic smartphone usage used as synonyms for this study?

Is the work clearly and accurately presented and does it cite the current literature?

Yes

If applicable, is the statistical analysis and its interpretation appropriate?

Yes

Are all the source data underlying the results available to ensure full reproducibility?

Yes

Is the study design appropriate and is the work technically sound?

Yes

Are the conclusions drawn adequately supported by the results?

Yes

Are sufficient details of methods and analysis provided to allow replication by others?

Partly

Reviewer Expertise:

Immunization, Adolescent health, Primary health care

I confirm that I have read this submission and believe that I have an appropriate level of expertise to confirm that it is of an acceptable scientific standard, however I have significant reservations, as outlined above.

# Response

**Published:** 2024-12-13

**Authors:** Jayashree K

**Affiliations:** Pediatrics, Manipal Academy of Higher Education, Mangalore, Karnataka, India

Major:

1. How was development of sexuality defined for this descriptive study: was it sexual orientation or sexual identity or something else?

**Author response:** Thank you for the comment. Sexuality has different components which includes biological sex, gender identity, social sex-role, and sexual orientation. In the present study we considered gender role identification, gender orientation as development of sexuality. The same has now been incorporated in the introduction section.

      2.Sexual awareness may begin at early adolescents (9-12 yrs), what was the reason authors tried to address or justify development of sexuality or influence of smartphone on sexuality at the age of 18-19 yrs ? Was this based on any observations or references or any other issues specific in your region?

**Author response:** Yes, it begins at early adolescence but there is lot sexual experimentation with respect to how they dress up and their activities/ likes/dislikes/ whom they hang out with/ gender roles etc, occur throughout the adolescent period and it keeps changing. But by the time they enter late adolescence a stable sexuality usually develops. Another reason why we didn’t take early adolescence is smartphone usage with respect to questions related to different components of sexuality may pose ethical issues also they are not mature enough to understand the questionnaire. The same has now been added to our Discussion section for further clarity.

             3.Discussion section mentions about friends/peers being the major source for information but results also show magazines and videos also which may be due to stigma to discuss with teachers, family or health professionals. Kindly elaborate this as it is relevant for the study objective with smartphone being the most easily handy and private source for all information for an adolescent.

**Author response: **Thank you for the suggestion. Our study found that friends/peers are the major source of information about pubertal changes followed by books/magazines as mentioned in the results section. We also found that only a small proportion of them turned to parents or health professionals for any information or concerns regarding the same. These findings aligned with other studies as described in the discussion section. As you rightly pointed out, smartphones, with their private nature, have emerged as a primary source of information for adolescents. This is especially true for sensitive topics where young people may feel uncomfortable engaging in open dialogue with adults. We have further elaborated on this in our discussion section.

 Minor:

  

1. Abstract mentions about "exposure to pornographic content with youngest being 9 years old" study subjects being late adolescents does this mean at the age of 9?

**Author response:** Thank you. It means the study subject who is a late adolescent started watching porn when he/she was 9 years old. The same has been mentioned in the sentence of Results section “In our study, 90% students had watched pornographic content and one of the students had watched when he was 9 years old…………………..”

             2.Are smartphone addiction and problematic smartphone usage used as synonyms for this study?

**Author response: **   Yes. We used the Smartphone Addiction Scale-Short Version (SAS-SV) to identify Problematic Smartphone Use (PSU) among study participants. Participants scoring 31 & above on the SAS-SV were considered to have problematic smartphone use. Throughout the study, the terms 'problematic smartphone use' and 'smartphone addiction' are used interchangeably. For better clarity, we have now mentioned this in the introduction section.
